# Supplementary material for: Causal Relationship between Aging and Anorexia Nervosa: A White-Matter-Microstructure-Mediated Mendelian Randomization Analysis
Source: Biomedicines. 2024 Aug 16;12(8):1874. doi: 10.3390/biomedicines12081874 (PMC11351342; doi:10.3390/biomedicines12081874)
Supplement: Supplementary file 1 [file biomedicines-12-01874-s001.zip › Supplementary Figure/Figure S9/Leaveoneout plot of MR analysis for ubm-b-1905 to AN.pdf]

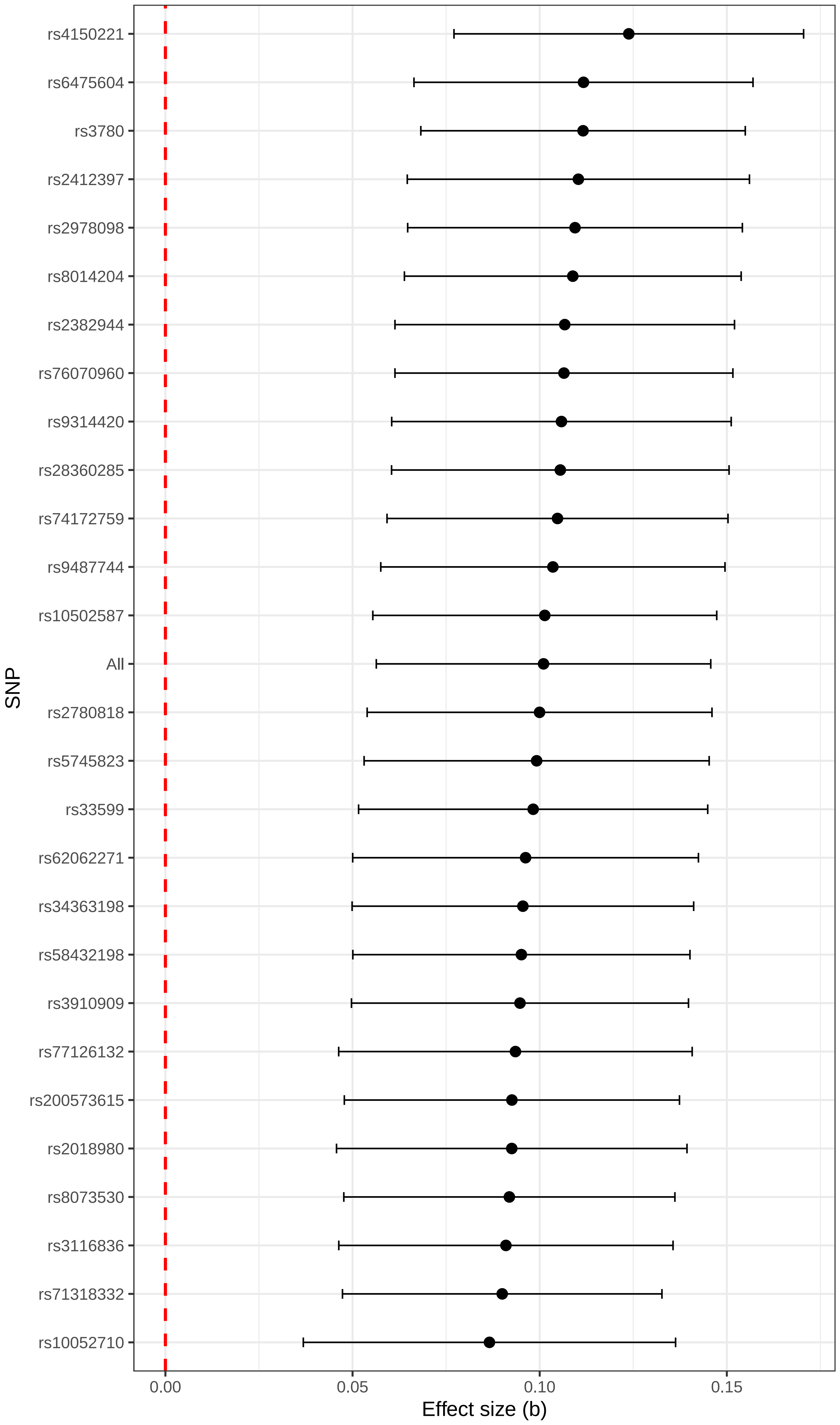
rs4150221
rs6475604
rs3780
rs2412397
rs2978098
rs8014204
rs2382944
rs76070960
rs9314420
rs28360285
rs74172759
rs9487744
rs10502587
All
rs2780818
rs5745823
rs33599
rs62062271
rs34363198
rs58432198
rs3910909
rs77126132
rs200573615
rs2018980
rs8073530
rs3116836
rs71318332
rs10052710
SNP
0.00
0.05
0.10
0.15
Effect size (b)
